# Deep learning-based generation of direct stopping power ratio maps for MR-only proton therapy of primary brain tumor patients

**DOI:** 10.1016/j.phro.2026.101034

**Published:** 2026-07-04

**Authors:** Emilie Alvarez-Michael, Nils Peters, Julian Schwengfelder, Daniel Zachow, Felix Raschke, Steffen Löck, Christian Richter, Rudi Apolle, Franciska Lebbink, Esther G.C. Troost

**Affiliations:** a OncoRay – National Center for Radiation Research in Oncology, Faculty of Medicine and University Hospital Carl Gustav Carus, TUD Dresden University of Technology, Helmholtz-Zentrum Dresden-Rossendorf, Dresden, Germany; b Department of Radiotherapy and Radiation Oncology, Faculty of Medicine and University Hospital Carl Gustav Carus, TUD Dresden University of Technology, Dresden, Germany; c Department of Radiation Oncology, University of Washington & Fred Hutch Cancer Center, Seattle, WA, USA; d Helmholtz-Zentrum Dresden-Rossendorf, Institute of Radiooncology – OncoRay, Dresden, Germany; e National Center for Tumor Diseases Dresden (NCT/UCC), Germany: German Cancer Research Center (DKFZ), Heidelberg, Germany; Faculty of Medicine and University Hospital Carl Gustav Carus, TUD Dresden University of Technology, Dresden, Germany; Helmholtz-Zentrum Dresden-Rossendorf (HZDR), Dresden, Germany; f German Cancer Consortium (DKTK), Partner Site Dresden, and German Cancer Research Center (DKFZ), Heidelberg, Germany

**Keywords:** Synthetic stopping power ratio, MRI, Deep learning, MR-only proton therapy

## Abstract

**Background and purpose:**

A magnetic resonance-only proton therapy (MRoPT) workflow requires synthetic computed tomography (CT) to derive the stopping power ratio (SPR) for treatment planning. Here, we introduce three U-Net models to predict synthetic SPR (sSPR) maps from magnetic resonance imaging (MRI) scans of primary brain tumor patients.

**Materials and methods:**

SPR maps and heterogenous MRI data were collected from 140 patients. Three 2D-U-Net models were independently developed with axial, coronal, and sagittal MRI scan slices, the combination of which resulted in 2.5D sSPR.

To evaluate the sSPR and associated dose maps, the mean absolute error (MAE), dose volume histogram-parameter differences, e.g. D2% and D98% of the clinical target volume (CTV), and the mean and maximum dose (Dmean and Dmax, respectively) for organs at risk (OAR; brainstem, optic chiasm, optic nerves, lacrimal glands and whole brain) were computed. Finally, gamma and range shifts analyses were performed.

**Results:**

The head cohort mean MAE was 0.062 ± 0.009 for the 2.5D sSPR. For the CTV, D2% and D98% differences ranged from −0.60% to 0.75%. Regarding the OAR, the Dmean and Dmax differences, corrected for relative biological effectiveness, were within ±2.70Gy (RBE). The mean pass rate for the local gamma criterion of 2%/2 mm with 10% dose threshold was 76.19 ± 4.67%. Averages of absolute range shifts ranged from 0.50 mm to 1.79 mm.

**Conclusions:**

Generated sSPR showed minor discrepancies from SPR. This study is a step closer to an MRoPT workflow for brain tumor patients, albeit the residual errors full impact needs to be more investigated in future work.

## Highlights

• Generation of synthetic stopping power ratio maps from magnetic resonance imaging.
• Development of a 2.5D U-Net model for predictions in primary brain tumor patients.
• High dosimetric agreement between synthetic and original stopping power maps.

## Graphical abstract

**Figure:**
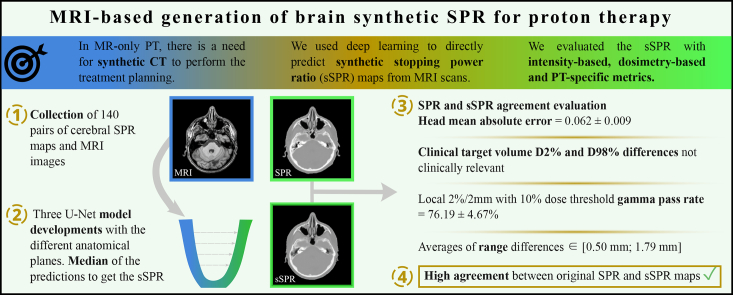

## 1 Introduction

In proton beam therapy (PT), computed tomography (CT) is the current clinical standard for treatment planning, since it holds anatomical and tissue density information obligatory for PT dose calculation. For target volume delineation of soft tissue tumors, such as primary brain tumors, tumors of the abdomen or pelvis, however, the CT needs to be fused with magnetic resonance imaging (MRI) scan in order to obtain a trustworthy, high-contrast depiction of the underlying anatomy. Since an MR-only workflow for photon therapy has already been established, an MR-only PT (MRoPT) workflow is gaining increasing interest [1], [2].

MRoPT holds many advantages over the conventional PT workflow: (1) it eliminates the patients' radiation dose originating from CT imaging, (2) it circumvents the geometric uncertainty caused by the CT-MRI image fusion, and (3) it streamlines the clinical workflow, ultimately lowering the treatment cost. The main obstacle for the introduction of MRoPT is the lack of quantitative tissue information, e.g., electron density or stopping power, in clinical MRI scans, since their grey values vary depending on pulse sequence, magnetic field strength and receiver coil sensitivity [3], [4], [5]. This makes direct dose calculations on MRI scans unfeasible. In contrast, CT provides geometrically accurate information on photon absorption from which quantitative information on tissue parameters like electron density can be derived [6]. For proton dose calculation, CT numbers (CTN) are converted into the corresponding tissue proton stopping power ratios (SPR) using a Hounsfield look-up table (HLUT). This translation accounts for a large part of the overall proton range uncertainty margin of (3.5% + 2 mm) [7], [8], [9].

Dual-energy CT (DECT)-based direct SPR prediction (DirectSPR) has been clinically implemented at several institutions, significantly reducing the uncertainty margin to as low as (1.7% + 2 mm) for PT of primary brain tumors [10]. The DirectSPR algorithm was extensively tested, validated, and subsequently introduced for patients with static tumors in our department in 2019 [11], [12], [13], [14], [15]. Approaches applied to other DECT implementations have demonstrated consistent results in directly calculating the SPR [16].

There are different approaches to create synthetic CT (sCT) images from MRI data for PT dose calculation, translating grey values into CTN via bulk density override, atlas-based attribution, applying voxel-based techniques, or using artificial intelligence (AI) [17]. The associated uncertainty, to which the aforementioned 3.5% uncertainty of translating CTN to SPR needs to be added, results in sCT maps of higher inaccuracy for PT dose calculation. Directly generating synthetic SPR (sSPR) maps from MRI scans, which has only been explored in a few studies, would circumvent the translation from CTN to SPR [18], [19], [20], [21], [22], [23], [24].

In this study, we therefore assessed the quality of the sSPR maps, which were directly obtained from MRI scans using deep learning (DL). We trained and validated a 2-dimensional (2D)-U-Net with pairs of MRI scans and SPR maps of primary brain tumor patients derived from DirectSPR. The DL models were developed with highly heterogeneous MRI data, i.e. different vendors, field strengths and imaging protocols. The quality of the sSPR maps in the test set was evaluated regarding their absolute accuracy as well as regarding their structural integrity compared to the clinical SPR maps. Finally, this study was the first to evaluate the dosimetric impact of remaining deviations in primary brain tumor patients.

## 2 Materials and methods

### 2.1 Patient and imaging details

MRI scans and DirectSPR-based SPR datasets of 140 primary brain tumor patients treated with adjuvant PT in the context of the prospective clinical trial „ProtoChoice-Hirn“(NCT02824731) at the Department of Radiotherapy and Radiation Oncology of University Hospital Carl Gustav Carus, Dresden, Germany, were included. The study was approved by the Ethics Committee of the TUD Dresden University of Technology (BO-EK-72022023). All DECT scans were acquired on a SOMATOM Definition AS scanner (Siemens Healthineers, Erlangen, Germany), with a tube voltage combination of 80/140 kVp and a $\mathrm{CTD}I_{\mathrm{vol},16\mathrm{cm}}\approx 32\;\mathrm{mGy}$. In addition, 79 keV pseudo-monoenergetic CT images were reconstructed for contouring purposes. Reconstructions were performed with the quantitative kernels Q34 (*N* = 120 patients) or Qr40 (*N* = 20 patients), depending on the software version, and SAFIRE at medium strength (3). These two kernels resulted in images sufficiently similar for the purpose of the study. A bone beam hardening correction was additionally applied [25]. The SPR maps were obtained using the DirectSPR application^1^ and had an original voxel size of 0.98 × 0.98 × 2 mm^3^.

### 2.2 Network architecture and training strategies

Details on the image pre-processing are provided in the Supplementary Material. 38 pairs of SPR/T1-weighted (T1w) MRI images and 60 pairs of SPR/contrast-enhanced T1w (T1wCE) MRI images were assigned to the training set. The validation set, used in parallel to the training set to fine-tune the network hyperparameters, consisted of 8 SPR/T1w-MRI images and 13 SPR/T1wCE-MRI image pairs. The remaining pairs of 8 SPR/T1w-MRI images and 13 SPR/T1wCE-MRI images were assigned to the test set. The 2D-U-Net used was similar to the architecture introduced by Ronneberger et al. [26], except for the use of a single kernel for the last convolution, 2D transposed convolutions, and dropout. A complete description is available in the Supplementary Material. The network was trained using the Adam optimizer, with a learning rate of 0.0005 [27] and a batch size of 16. The mean absolute error (MAE) was selected as loss function. Early stopping was implemented on the validation set, with a patience of 25 [28]. All DL model computations were performed with Keras from Tensorflow-gpu (version 2.4.1) on a NVIDIA Tesla V100 PCIe 16 GB.

The network was trained with either axial, coronal, or sagittal slices, resulting in three different models. For each patient, the voxel-wise median of these three resulting sSPR maps was computed, to obtain a 2.5D sSPR, similarly to Dinkla et al. [29].

### 2.3 Quantitative image evaluation

To assess the voxel-wise agreement between SPR and sSPR, the MAE and the mean error (ME) were computed within the head masks. Additionally, three different tissue regions, i.e. air (SPR ≤ 0.85), bone (SPR ≥ 1.2), and soft tissues (1.02 ≤ SPR ≤ 1.06) were evaluated by applying the respective thresholds to the head masks.

Furthermore, the normalized MAE (NMAE) [30], which aims to evaluate the MAE error relative to the ground-truth intensity, was computed in the different tissue regions using:

$$\mathrm{NMAE}=\frac{1}{I}\sum_{i=1}^{I}\frac{|{\mathrm{SPR}}_{i}-{\mathrm{sSPR}}_{i}|}{{\mathrm{SPR}}_{i}}$$

with *I* being the number of voxels in the region of interest, *SPR*_*i*_ and *sSPR*_*i*_ the intensity of voxel *i* in the SPR and sSPR, respectively.

The peak signal-to-noise ratio (PSNR), expressed in decibels (dB), penalizes sSPR mis-predicted areas more than the MAE, ME and NMAE, owing to the mean squared error present at the denominator fraction. It was evaluated in the head masks. Structural integrity within the head masks was evaluated using the structural similarity index measure (SSIM). For bone, Dice similarity coefficients (DSC) were calculated as a measure of the volumetric overlap between the SPR and sSPR.

### 2.4 Dosimetric and proton range evaluations

Clinically applied PT treatment plans using the pencil beam scanning technique, which had been optimized with 1.7% + 2 mm range uncertainty, isotropic 3 mm position uncertainty, and 3 × 3 × 3 mm^3^ dose voxel size, were used for dosimetric evaluations. For this, the treatment plans were re-calculated without re-optimization on the SPR and sSPR using RayStation 11B-R (RaySearch Laboratories, Stockholm, Sweden) and a Monte Carlo dose engine with an uncertainty of 0.3%. For different structures, the dose volume histogram (DVH)-based differences were calculated as *D*_X%_ corresponding to the dose in X% of the structure. This included *D*_2%_ and *D*_98%_ for the clinical target volume (CTV); maximum dose (*D*_max_) for the organs at risk (OAR) brainstem, optic chiasm, optic nerves, and lacrimal glands; and mean dose (*D*_mean_) for the whole brain. The DVH differences were corrected for relative biological effectiveness and were expressed in Gy (RBE). To evaluate the sSPR and SPR dosimetric agreement, a gamma analysis was performed. For this, pass rates for the global and local 2%/2 mm and 3%/3 mm gamma criteria with 10% and 90% dose thresholds were computed with 3D Slicer (version 5.2.2). To investigate the actual influence on proton range during treatments, differences in water equivalent proton range were assessed between original SPR and sSPR. For this, proton ranges corresponding to 80% of the absorbed dose from the beam's-eye-view direction were computed with an in-house algorithm. For every patient, the beam-wise absolute range differences and associated averages were investigated. Dose homogeneity indices (HI) were computed for both original SPR and sSPR to assess the target coverage differences, according to:

$$\mathrm{HI}=\frac{D_{2\%}-D_{98\%}}{D_{\mathrm{mean}}}$$

### 2.5 Statistical analyses

For the four sSPR generation strategies (axial, coronal, sagittal and 2.5D), Wilcoxon signed-rank tests were performed with SciPy (version 1.10.1) to assess the significance of the image quality-based metric differences. For this, two sSPR approaches were selected, without order, which resulted in six pairs to be tested. To limit the type I error occurrence due to multiple testing, the significance level of 0.05 was corrected as per Bonferroni to a value of 0.008 [31].

## 3 Results

For all patients [median age 54.5 years (range: 18.0–86.0 years)], either T1w- (54 patients) or T1wCE- (86 patients) MRI images with pixel sizes of 0.34–1.0 mm and slice thicknesses of 1.0–3.0 mm, acquired on ten different MR scanners, were retrieved (Table S3). The MRI scans were postoperatively obtained for PT planning purposes, with a median interval between the acquisitions of the DECT and MRI scans of 0 days (range: 0–41 days). The characteristics of the test set patients, which were used as reference for all evaluations, are described in Table 1. The prescribed PT doses were 30 Gy (RBE) (*N* = 1 patient; re-irradiation case), 50 Gy (RBE) (N = 1), 54 Gy (RBE) (*N* = 4), and 60 Gy (RBE) (*N* = 15 patients), all delivered in 2 Gy (RBE) fractions.

**Table 1:** Characteristics of the test set patients.

Patient number	Tumor type	Grade	CTV (cm**^3^**)	Prescribed dose (Gy (RBE))	Number of beams
1	Astrocytoma	III	17.69	30	2
2	Astrocytoma	III	73.21	60	2
3	Glioblastoma Multiforme	IV	71.42	60	3
4	Glioblastoma Multiforme	IV	80.22	60	3
5	Glioblastoma Multiforme	IV	40.88	50	2
6	Astrocytoma	III	73.87	60	3
7	Oligodendroglioma	IV	260.08	60	3
8	Glioblastoma Multiforme	IV	175.22	60	3
9	Hemangiopericytoma	IIa	8.17	60	2
10	Glioblastoma Multiforme	IV	39.47	60	3
11	Astrocytoma	III	35.58	60	3
12	Glioblastoma Multiforme	IV	92.59	60	3
13	Glioblastoma Multiforme	IV	99.32	60	2
14	Astrocytoma	II	76.47	60	2
15	Astrocytoma	III	81.91	60	3
16	Astrocytoma	II	40.15	54	3
			152.11		
17	Glioblastoma Multiforme	IV	38.43	60	2
18	Glioblastoma Multiforme	IV	34.81	60	2
19	Meningioma	I	90.57	54	2
20	Astrocytoma	II	105.35	54	2
21	Meningioma	I	48.74	54	3

Abbreviations: CTV: clinical target volume; cm**^3^**: cubic centimetre; Gy: Grey; RBE: relative biological effectiveness.

The training in the axial, coronal, and sagittal directions required 5.6, 4.6, and 4.0 h, respectively. Generating the sSPR for the whole test set took 24 s.

### 3.1 Quantitative image evaluation

**Fig. 1:**
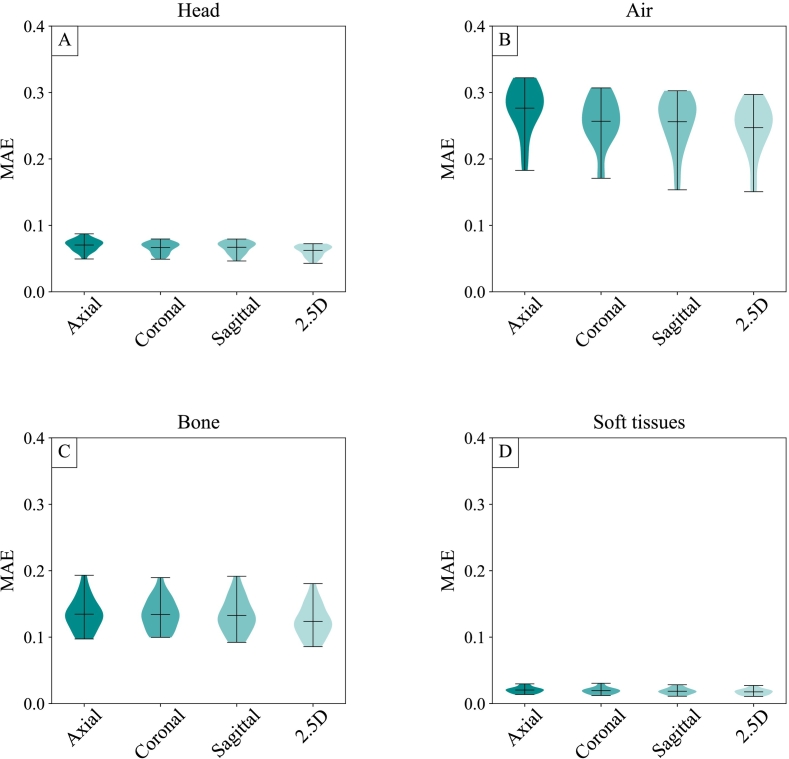
Mean absolute error (MAE) for the four different head regions: head (A), air (B), bone (C) and soft tissues (D). The black lines in the violin plots correspond to the minima, maxima, and means of the distributions.

The 2.5D sSPR presented the best performance in the head, with the lowest MAE (*p* < 0.001 for the three tests; Fig. 1). In addition, this approach resulted in the highest PSNR (27.98 ± 1.23 dB) and SSIM (0.89 ± 0.02; *p* < 0.001 for the six tests in total; Table 2). Moreover, the 2.5D approach achieved the best performance in bone, with the lowest MAE (0.124 ± 0.025), lowest NMAE (8.2 ± 1.4%), and highest DSC (0.86 ± 0.03; *p* < 0.001 for the nine tests in total). Patient-specific MAE performances are given in Table S4.

**Table 2:** MAE, NMAE, ME, PSNR, SSIM and DSC of the four different synthetic stopping power ratio map approaches. Mean ± standard deviations are presented. The metrics were computed on the test set patients.

Metric	Evaluated region	Axial	Coronal	Sagittal	2.5D
MAE	Head	0.070 ± 0.009	0.067 ± 0.008	0.067 ± 0.009	0.062 ± 0.009
	Air	0.277 ± 0.037	0.257 ± 0.037	0.256 ± 0.040	0.247 ± 0.039
	Bone	0.135 ± 0.025	0.134 ± 0.024	0.133 ± 0.026	0.124 ± 0.025
	Soft tissues	0.020 ± 0.005	0.019 ± 0.005	0.019 ± 0.005	0.018 ± 0.004
NMAE (%)	Head	20.0 ± 27.2	14.3 ± 24.0	23.8 ± 32.3	18.0 ± 26.4
	Air	251.0 ± 435.8	153.8 ± 393.2	307.9 ± 487.2	227.4 ± 423.8
	Bone	9.0 ± 1.4	8.9 ± 1.3	8.8 ± 1.5	8.2 ± 1.4
	Soft tissues	2.0 ± 0.4	1.9 ± 0.5	1.8 ± 0.5	1.7 ± 0.4
ME	Head	0.015 ± 0.010	0.022 ± 0.010	0.020 ± 0.010	0.019 ± 0.010
	Air	−0.069 ± 0.060	−0.058 ± 0.058	−0.052 ± 0.063	−0.054 ± 0.058
	Bone	0.058 ± 0.037	0.084 ± 0.034	0.079 ± 0.037	0.071 ± 0.035
	Soft tissues	0.010 ± 0.004	0.012 ± 0.004	0.009 ± 0.004	0.010 ± 0.004
PSNR (dB)	Head	27.28 ± 1.01	27.55 ± 1.12	27.42 ± 1.16	27.98 ± 1.23
SSIM	Head	0.87 ± 0.02	0.87 ± 0.02	0.87 ± 0.02	0.89 ± 0.02
DSC	Bone	0.83 ± 0.04	0.84 ± 0.03	0.84 ± 0.04	0.86 ± 0.03

Abbreviations: MAE: mean absolute error; NMAE: normalized mean absolute error; ME: mean error; PSNR: peak signal-to-noise ratio; dB: decibel; SSIM: structural similarity index measure; DSC: Dice similarity coefficient.

**Fig. 2:**
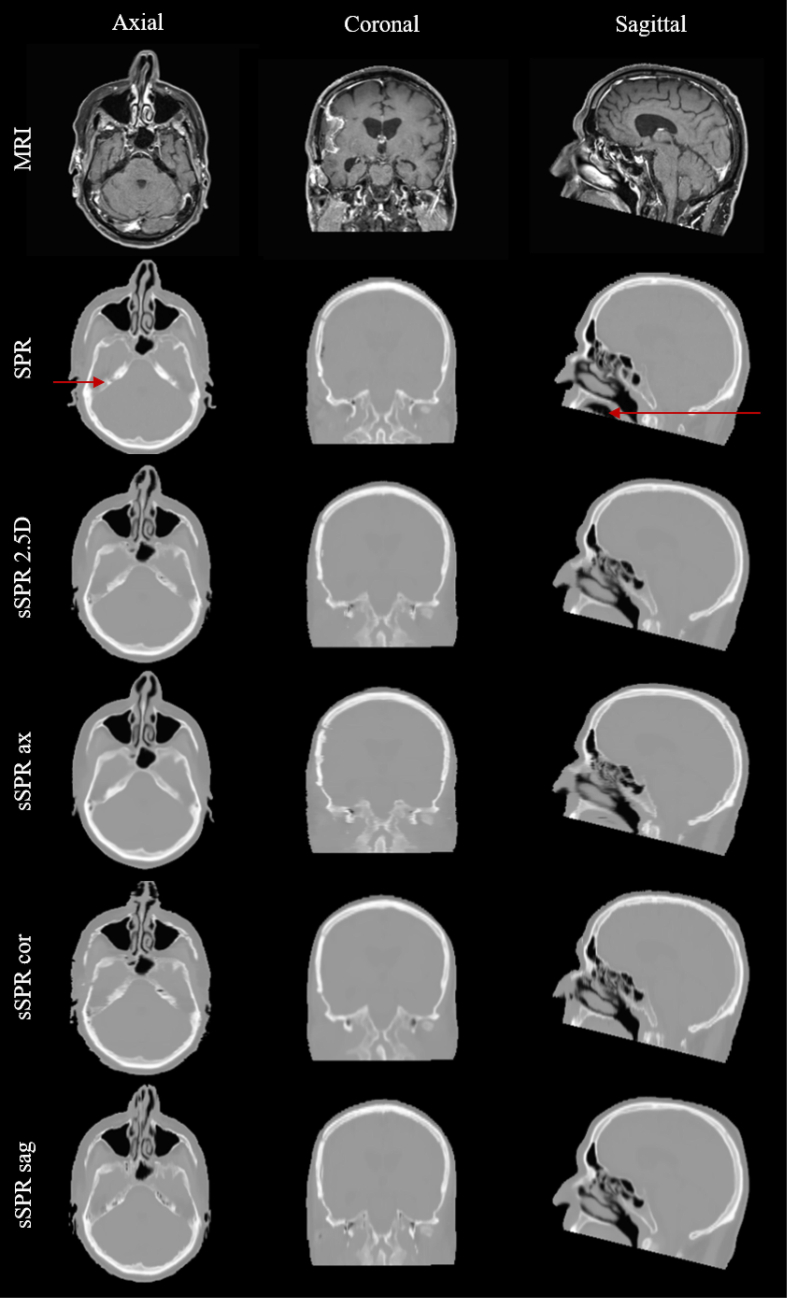
Axial, coronal, and sagittal views of patient 14. Rows show the contrast-enhanced T1-weighted magnetic resonance imaging (MRI) scan, original stopping power ratio (SPR) map, and the synthetic SPR (sSPR) maps obtained from the four approaches. The red arrows highlight regions from the original SPR that were incorrectly reconstructed in the sSPR. Window width/level: [1735 HU* / -156 HU*]. (For interpretation of the references to colour in this figure legend, the reader is referred to the web version of this article.)

An example of the sSPR generation from a T1wCE-MRI scan is shown in Fig. 2 for patient 14, in whom the intensity-based performance was somewhat better than on average. In detail, for the head MAE, the 2.5D, axial, coronal and sagittal sSPR approaches resulted in errors of 0.057, 0.063, 0.062 and 0.060, respectively. The temporal bone was incorrectly reconstructed by all approaches, and the air in the oral cavity was neither visible on the input MRI image nor on the derived sSPR, even though present on the SPR. Similar values were obtained for the sSPR generation from a T1w-MRI image (Fig. S5).

### 3.2 Dosimetric and proton range evaluations

Dosimetric analyses were performed on the 2.5D approach datasets due to their significantly better performance regarding bone MAE, bone NMAE and DSC (*p* < 0.001 for the nine tests in total). The *D*_2%_ and the *D*_98%_ of the sSPR in the CTV differed from the value obtained using the SPR method by up to [−0.60% - 0.75%] (Fig. S6). For the OAR, *D*_max_ differences were up to −2.70 Gy (RBE) for the left lacrimal gland, and 1.66 Gy (RBE) for the right lacrimal gland. The *D*_mean_ differences for the whole brain ranged between −0.25 Gy (RBE) and 0.11 Gy (RBE) (Fig. S6).

The sSPR and SPR high dose regions had a mean pass rate of 99.13% (Table 3; local 2%/2 mm gamma index with 90% dose threshold). This same gamma index resulted in a mean pass rate of 76.19% for the 10% dose threshold, i.e. the low dose region.

**Table 3:** Gamma indices of the 2.5D synthetic stopping power ratio approach. The metrics were computed on the test set.

Pass rates (%)	Global	10% dose threshold	2%/2 mm	90.70 ± 3.87
			3%/3 mm	95.08 ± 2.66
		90% dose threshold	2%/2 mm	99.29 ± 1.39
			3%/3 mm	99.79 ± 0.64
	Local	10% dose threshold	2%/2 mm	76.19 ± 4.67
			3%/3 mm	82.08 ± 4.03
		90% dose threshold	2%/2 mm	99.13 ± 1.58
			3%/3 mm	99.72 ± 0.77

The averages of the absolute water equivalent range differences varied between 0.50 mm to 1.79 mm, with individual beam-wise absolute differences [5th percentile; 95th percentile] of [0.03 mm; 6.78 mm] for the whole test set.

**Fig. 3:**
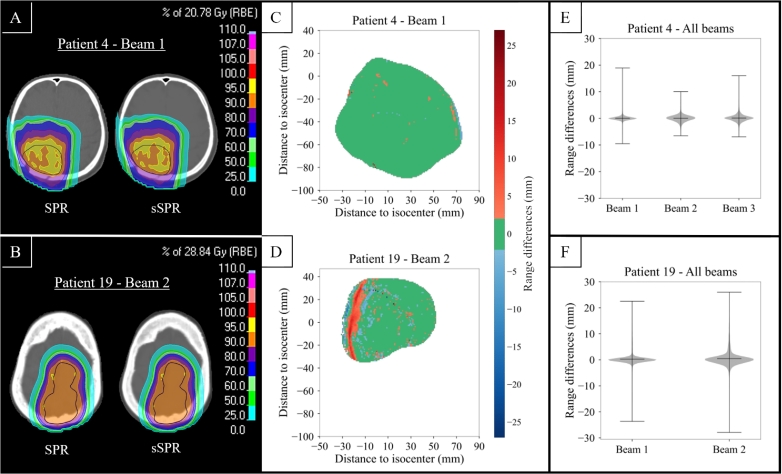
Stopping power ratio (SPR) and synthetic SPR (sSPR) dose maps (Fig. 3A and B), water equivalent thickness differences maps (Fig. 3C and D) and violin plots (Fig. 3E and F) of patient 4 and patient 19, with low and high averaged absolute range differences, respectively. The dark blue lines on the dose maps (Fig. 3A and B) represent the clinical target volumes. The black lines in the violin plots correspond to the minima, maxima, and means of the distributions. Window width/level: [750 HU* / 100 HU*]. (For interpretation of the references to colour in this figure legend, the reader is referred to the web version of this article.)

Patients with small (Patient 4) and large (Patient 19) averaged absolute shifts are depicted in Fig. 3. For patient 4, averaged absolute shift differences of 0.50 mm, 0.91 mm and 0.90 mm for beams 1, 2 and 3 were obtained, while for patient 19, these values were 1.05 mm and 1.73 mm for beams 1 and 2, respectively. Deviations were mostly located at the periphery of the high dose region (Fig. 3D). No structural difference was observed between the SPR and sSPR dose maps (Fig. 3A and B).

Lastly, the HI for the treatment plans generated with the original SPR and sSPR generated by the 2.5D approach showed no significant difference (0.06 ± 0.04).

## 4 Discussion

On the path of developing an MRoPT workflow, a DL approach was used in this study to derive sSPR maps from T1w- and T1wCE-MRI images of primary brain tumor patients. Datasets were evaluated using quantitative and structural imaging metrics as well as PT-specific metrics, such as the water equivalent range differences between original SPR and sSPR, and dosimetric metrics. Overall, promising sSPR quality was achieved, demonstrated by the acceptable MAE for the 2.5D approach, and the similarity in the predicted dose deposition between SPR and sSPR. To our knowledge, this is one of the few studies that investigated the direct translation of brain MRI images into SPR maps [18], [21]. In contrast to the other publications, our MR acquisition parameters substantially varied, and images obtained on several MR devices were included. Most importantly, we were the first to perform detailed dosimetric analyses.

The 2.5D head MAE, computed before the SPR to CTN conversion process, was 62 ± 9 HU*, which exceeded that of other investigations. Wang et al. [21] collected T1w- and T2 weighted (T2w)-MRI scans as well as T2-weighted fluid-attenuated inversion recovery (FLAIR) MRI scans from 195 brain tumor patients. Using a cycle-consistent generative adversarial network (GAN), they computed synthetic relative SPR. Without pre-processing, the T1w-MRI, T2w-MRI, T2w-FLAIR MRI scans, and a combination of all MR sequences resulted in MAE of 50 ± 18 HU, 54 ± 21 HU, 59 ± 22 HU, and 83 ± 29 HU, respectively, demonstrating that multiple channel MRI scans as input leads to significantly higher MAE than considering only individual MRI scans. In our study however, T1w- and T1wCE-MRI scans were chosen as single channel network input, since a previous study on sCT for photon therapy reported non-significant dosimetric differences between these two MR sequences [32]. Recently, Omidi et al. [18] developed a 2D cycle-consistent GAN to predict synthetic SPR from T1-weighted MRI scans. They retrieved 5 and 5 brain tumor patients to train and test the network, respectively. The ground-truth SPR maps were obtained with a HLUT approach. They achieved a head MAE of 1.13%, which may be compared with caution to our mean head NMAE for the coronal approach of 14.3%. However, their small dataset may have introduced biases during the model evaluation.

The analysis of our tissue-region-specific ME showed that air and bone sSPR intensity predictions were over- and under-estimated, respectively, leading to under- and overestimations of the proton ranges, depending on the tissue traversed. In our study, air and bone ME of −54 ± 58 HU* and 71 ± 35 HU* were obtained for the 2.5D approach, after conversion to CTN. For the T1w-MRI scans pre-processed with N4 and histogram matching, Wang et al. [21] applied thresholds of −800 HU and 200 HU to compute their air and bone ME of 22 ± 14 HU and 89 ± 13 HU, respectively. Since they used the same bone threshold as in our study, one could directly conclude that we outperformed their bone ME. Regarding air, they used a threshold significantly lower than ours, resulting in the evaluation of a smaller number of voxels which could justify their lower ME in this region.

The CTV and OAR dosimetric differences described here were small and within the given tolerance limits of our clinic. After visually examining two patients with increased dose to the lacrimal glands, it was noticed that a mis-registration had occurred in the frontal and nasal bony regions, negatively impacting the reconstruction of the lacrimal gland.

In our study, we found higher range differences at the periphery of the presented map in beam's eye view, i.e. in the dose gradient regions. As a result, the dose agreement between original SPR and sSPR should be assessed by the local 2%/2 mm criterion with 10% dose threshold, since this considers these specific dose gradient errors. Here, a mean pass rate of 76.19% was obtained, leaving room for future improvement. To our knowledge, no dosimetric gamma index analysis has been performed in the previous sSPR literature using patient data. Averages of beam-wise absolute range shifts up to 1.8 mm were obtained, which was larger than reported values for brain sCT of 1.1 ± 0.9 mm [33]. In one patient (patient 19), high range differences were found (Fig. 3F), which turned out to only have a limited impact on the *D*_2%_ and *D*_98%_ differences in the CTV (deviations of −0.16% and 0.52%, respectively).

The main advantage of our approach is the use of SPR directly obtained with DECT as ground truth for sSPR generation, which ensures the comparison with the currently highest-achievable SPR accuracy. Indeed, Peters et al. [10] reported the prediction accuracy of this method with a 1σ uncertainty of 1.30%, 1.62% and up to 1.30% for lung, bones, and soft tissues, respectively. An additional strength of our work is the large variety of MR acquisition parameters (at least six sequences, and five slice thicknesses), and the inclusion of MRI images from ten different MR scanners. This ensures a high robustness and generalizability of the developed models.

Although our study holds promising findings, there are some limitations. First, the non-simultaneous acquisition of DECT and MRI scans resulted in potential anatomical changes in between scan acquisitions. To tackle this shortcoming, we split the test set into two subsets of patients having received both scans within a day and those with more time between scans. The latter subgroup only resulted in significantly poorer performances for the averages of absolute range differences metric (Mann-Whitney *U* test, *p* = 0.006 for the one test; Fig. 2 as an example of a patient with a tongue movement). Second, the reduced number of patients attributed to the test set may have compromised our findings. A larger cohort, with more non-standard patients, would have further improved the model performance and its generalizability. However, to our knowledge, the 140 patients included in this study were the second largest cohort among the studies on MRI scan-derived sSPR generation with AI. Lastly, the DL model included several uncertainty and error sources, deriving from the potential MRI scan geometric distortions [34], the MRI scan to SPR map registrations [35] and the DECT scan to SPR map conversions [10].

To conclude, sSPR, trained with DirectSPR datasets obtained from DECT, were successfully generated from conventional T1w-MRI scans (with or without contrast agent) with DL. The quality of the sSPR maps was confirmed by the low MAE, the small dosimetric differences and the small beam range shift averages. This study is a step closer to a MRoPT workflow for brain tumor patients, albeit the full impact of residual errors needs to be evaluated more comprehensively in future work.

## CRediT authorship contribution statement

**Emilie Alvarez-Michael:** Writing – review & editing, Writing – original draft, Software, Methodology, Investigation, Formal analysis, Data curation, Conceptualization. **Nils Peters:** Writing – review & editing, Software, Methodology, Conceptualization. **Julian Schwengfelder:** Writing – review & editing, Software, Methodology. **Daniel Zachow:** Software, Data curation. **Felix Raschke:** Writing – review & editing, Software, Conceptualization. **Steffen Löck:** Writing – review & editing, Resources, Methodology. **Christian Richter:** Writing – review & editing, Conceptualization. **Rudi Apolle:** Writing – review & editing. **Franciska Lebbink:** Writing – review & editing, Writing – original draft, Methodology, Formal analysis. **Esther G.C. Troost:** Writing – review & editing, Writing – original draft, Supervision, Methodology, Conceptualization.

## Declaration of competing interest

The authors declare the following financial interests/personal relationships which may be considered as potential competing interests: OncoRay has an institutional research agreement with Siemens Healthineers in the field of CT imaging for particle therapy. Furthermore, OncoRay has an institutional agreement as reference center for dual-energy CT in radiotherapy as well as a software evaluation contract with Siemens Healthineers. For the present study, the authors received no financial support involved in the study design or materials used, nor in the collection, analysis and interpretation of data nor in the writing of the publication. Prof. Esther G. C. Troost is a member of the Scientific Advisory Board of IBA.

## Appendix A Supplementary data

Supplementary_file_Alvarez-Michael_MRI_sSPR_DL.

## Footnotes

^1^ SPR values were stored in a scaled fashion similar to CTN to meet DICOM format requirements and ease interpretation. SPR maps were calculated as $\mathrm{SP}R_{\mathrm{scaled}}=\left(\mathrm{SPR}-1\right)*1000$, such that values of 0 HU* and − 1000 HU* corresponded to water and air, respectively. HU* is used, as the information is stored in DICOM-compatible CT number format but contains SPR information instead of X-ray attenuation.
