# Supplementary material for: Deep learning-based generation of direct stopping power ratio maps for MR-only proton therapy of primary brain tumor patients
Source: Phys Imaging Radiat Oncol. 2026 Jul 4;40:101034. doi: 10.1016/j.phro.2026.101034 (PMC13416828; doi:10.1016/j.phro.2026.101034)
Supplement: Supplementary file 1 — Supplementary_file_Alvarez-Michael_MRI_sSPR_DL. [file mmc1.pdf]

Supplementary material

Description S1: Image pre-processing

Binary head masks were first created for SPR data with a lower cut-off of SPR = 0.75 and for MRI data using Otsu binning (library MedPy 0.4.0). To further improve the foreground/background assignment, masks were adapted with dilation and erosion morphological operations to close the binary images, hole filling, labelling and convolutions. The application level of these operations was mainly slice-wise, and for all three different image views. To homogenize the image backgrounds, the regions outside the head mask were set to air-equivalent values (CTN = -1024 HU* in the SPR maps and intensity = 0 in the MRI scans). A N4-based filter (Simple ITK version 2.2.0) was applied to the MRI data for bias field correction. Based on a linear interpolation, both the SPR maps and MRI scans were spatially resampled to $1 \times 1 \times 1$ mm^3, to ensure that all input data for the neural network had the same voxel size. The MRI scans were then rigidly registered to the SPR maps using the ANTsPy library (version 0.4.2). The derived transformations were applied to the MRI scans and associated masks. Based on padding and cropping operations, image sizes were harmonized to $256 \times 256 \times 256$, to meet neural network architecture constraints. To limit the impact of the MRI scan intensity variability, the latter was normalized with a Z-score approach that set the intensity mean and variance of each MRI scan to 0 and 1, respectively. Lastly, to improve the training stability, SPR datasets were globally rescaled to [-1; 1] with cohort-based minimum and maximum of -1024 HU* and 3071 HU*, respectively.

Description S2: Network architecture

Regarding the 2D network architecture used, the encoding path was constituted of 4 different blocks. Each block was composed of a series of a convolution layer, drop-out layer, convolution layer and max pooling layer. The numbers of kernels of the convolution layers were respectively equal to 64, 128, 256 and 512 for blocks 1, 2, 3 and 4. All convolution layers presented a kernel size of 3×3. For the max pooling layers, the windows size was of size 2×2. The drop-out rates were equal to 0.1 for the two first blocks, and to 0.2 for the two last blocks.

The network base was composed of a convolutional layer, drop-out layer and convolutional layer. The two convolution layers presented 1024 kernels of size 3×3. The drop-out rate was equal to 0.3.

The decoding path was composed of 4 blocks. Each block contained a series of transposed convolution layer, concatenation between the output of the latter transposed convolution and the second convolution layer output of the symmetric encoding block, convolution layer, drop-out layer, and convolution layer. The numbers of kernels of the transposed convolution and convolution layers were the same as the symmetric encoding path blocks, i.e. equal to 64, 128, 256 and 512. The kernel sizes were 2×2 and 3×3 for the transposed convolution and convolution layers respectively. The drop-out rates also corresponded to the symmetric encoding blocks, i.e. equal to 0.1 and 0.2.

Note that all convolution layers previously introduced presented the rectified linear unit as activation function.

The final layer was a convolution with one kernel of size 1×1 and a linear activation function.

Table S3: Description of the MRI data.

			Number of patients
T1w-magnetic resonance imaging	MR device	Ingenuity TF PET/MR 3 T (Philips Healthcare, Best, Netherlands)	48
		Skyra 3 T (Siemens Healthineers, Erlangen, Germany)	2
		SIGNA HDxt 1.5 T (GE Healthcare, Chicago, Illinois)	2
		Lumina 3 T (Siemens Healthineers, Erlangen, Germany)	1
		Verio Dot 3 T (Siemens Healthineers, Erlangen, Germany)	1
	Sequence	Ultrafast gradient echo	47
		Fast spin echo	3
		Ultrafast spoiled gradient echo	2
		Ultrafast 3D gradient echo	1
		NA	1
	Repetition time (ms)	7.91	1
		[8; 9]	48
		10	1
		[550; 570]	2
		800	1
		2300	1
	Echo time (ms)	[3; 4]	51
		[9; 10]	2
		11	1
	Flip angle (°)	8	48
		12	2
		20	1
		132	1
		150	2
		0.69	1

	**Pixel size (mm)**	[0.85; 0.90]	2
		[0.95; 1]	51
	**Slice thickness (mm)**	1	47
		1.6	2
		3	5
**Contrast-enhanced T1w magnetic resonance imaging**	**MR device**	Vida 3 T (Siemens Healthineers, Erlangen, Germany)	32
		Ingenuity TF PET/MR 3 T (Philips Healthcare, Best, Netherlands)	18
		Verio Dot 3 T (Siemens Healthineers, Erlangen, Germany)	14
		Skyra 3 T (Siemens Healthineers, Erlangen, Germany)	9
		Verio 3 T (Siemens Healthineers, Erlangen, Germany)	8
		SIGNA HDxt 1.5 T (GE Healthcare, Chicago, Illinois)	2
		Magnetom Aera 1.5 T (Siemens Healthineers, Er langen, Germany)	1
		Avanto 1.5 T (Siemens Healthineers, Erlangen, Germany)	1
		Prisma 3 T (Siemens Healthineers, Erlangen, Germany)	1
	**Sequence**	3D fast spin echo	45
		Ultrafast gradient echo	18
		Ultrafast 3D gradient echo	16
		Fast spin echo	4
		Ultrafast spoiled gradient echo	2
		3D spoiled gradient echo	1
	**Repetition time (ms)**	6.17	1
		[8; 9]	20
		20	1

		[550; 650]	3
		[700; 850]	46
		1800	8
		[2200; 2300]	7
	**Echo time (ms)**	[2; 3]	9
		[3; 4]	28
		[9; 10]	3
		11	1
		17	1
		19	31
		23	13
	**Flip angle (°)**	8	25
		10	1
		12	11
		120	45
		150	4
	**Pixel size (mm)**	0.34	1
		0.53	1
		0.69	2
		[0.85; 0.90]	2
		[0.95; 1]	80
	**Slice thickness (mm)**	1	76
		1.1	1
		1.6	2
		2	1
		3	6

Table S4: Patient-specific performances for the mean absolute stopping power ratio error computed on the test set.

Patient number	Axial	Coronal	Sagittal	2.5D
**1**	0.075	0.067	0.069	0.063
**2**	0.071	0.068	0.067	0.064
**3**	0.078	0.075	0.075	0.071
**4**	0.072	0.074	0.070	0.067
**5**	0.075	0.071	0.074	0.068
**6**	0.069	0.066	0.065	0.062
**7**	0.087	0.076	0.079	0.072
**8**	0.049	0.049	0.046	0.043
**9**	0.078	0.074	0.076	0.072
**10**	0.058	0.053	0.051	0.048
**11**	0.061	0.056	0.057	0.051
**12**	0.070	0.068	0.069	0.063
**13**	0.082	0.079	0.077	0.072
**14**	0.063	0.062	0.060	0.057
**15**	0.078	0.074	0.073	0.068
**16**	0.072	0.070	0.075	0.067
**17**	0.073	0.072	0.071	0.068
**18**	0.063	0.058	0.056	0.053
**19**	0.075	0.071	0.072	0.068
**20**	0.063	0.056	0.059	0.053
**21**	0.064	0.062	0.063	0.057

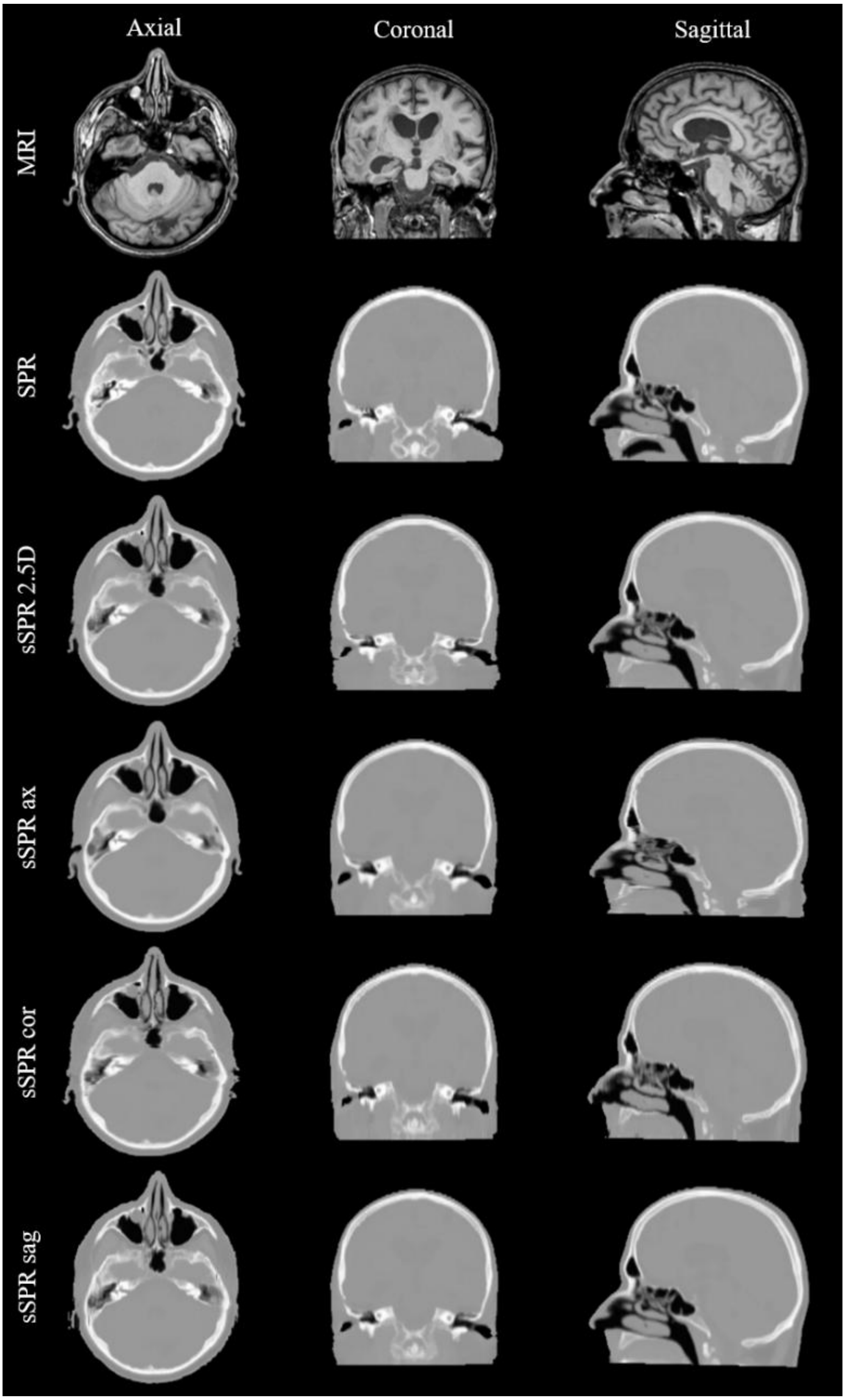

Figure S5: Axial, coronal, and sagittal views of patient 21. Rows show the T1-weighted magnetic resonance imaging (MRI) scan, original stopping power ratio (SPR) map, as well as synthetic SPR (sSPR) maps obtained from the four approaches at a window width/level: [1735 HU* / -156 HU*]. Visual inspection showed good concordance between the SPR and 2.5D sSPR, which was quantitatively confirmed.

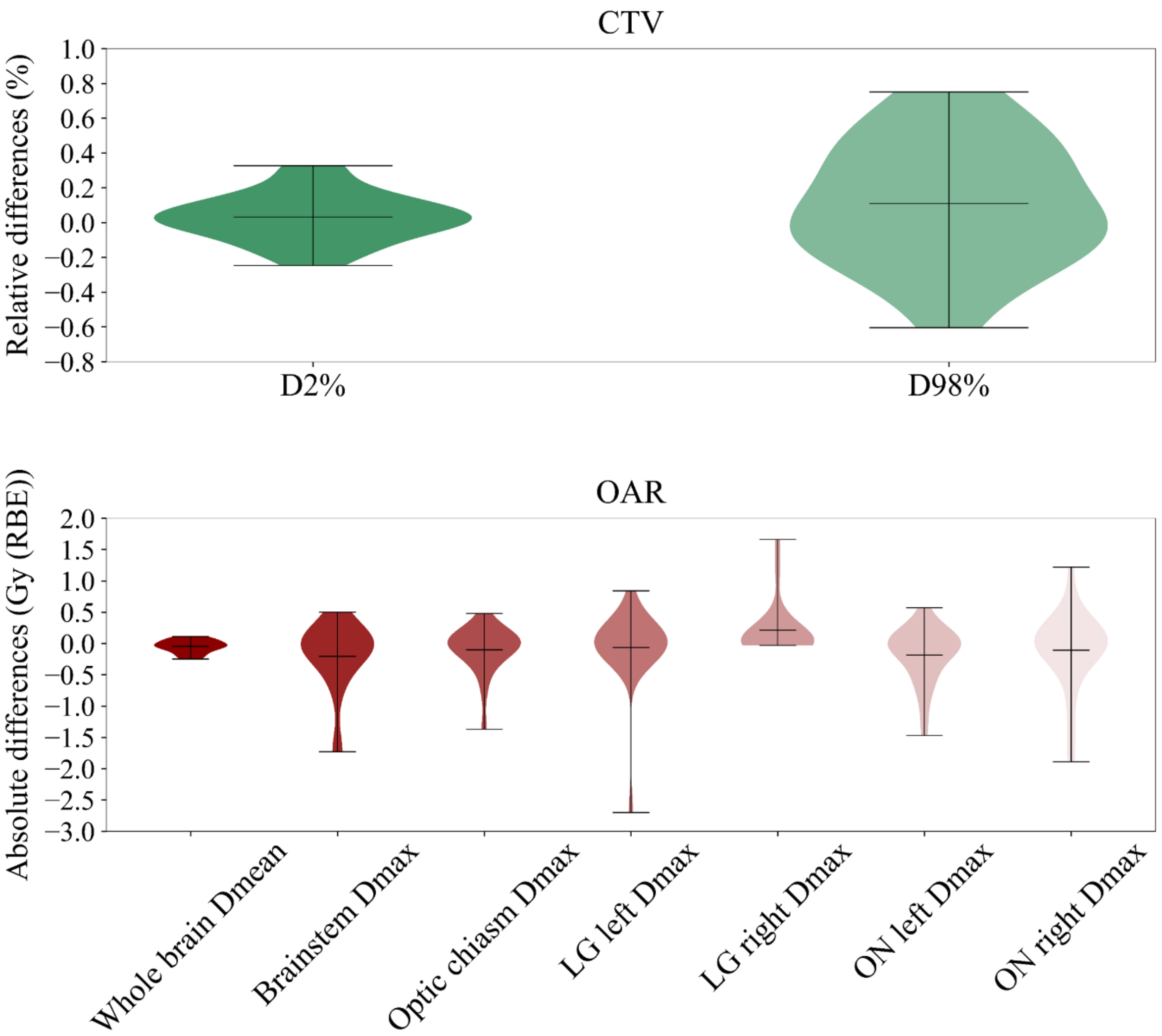

Figure S6: Dose volume histogram parameter differences for the clinical target volume (CTV; upper figure) and the organs at risk (OAR; lower figure) for all patients in the test set. The black lines in the violin plots correspond to the minima, maxima, and means of the distributions.

Abbreviations: D2%: dose in 2% of the volume; D98%: dose in 98% of the volume; Dmean: mean dose; RBE: relative biological effectiveness; Dmax: maximum dose; LG: lacrimal gland; ON: optic nerve.
